# Familial cancer among consecutive uterine cancer patients in Sweden

**DOI:** 10.1186/1897-4287-12-14

**Published:** 2014-05-07

**Authors:** Gerasimos Tzortzatos, Ofra Wersäll, Kristina Gemzell Danielsson, Annika Lindblom, Emma Tham, Miriam Mints

**Affiliations:** 1 Department of Women’s and Children’s Health, Division of Obstetrics and Gynecology, Karolinska Institutet, Karolinska University Hospital, Solna/Huddinge, Stockholm, Sweden; 2 Department of Clinical Genetics, Karolinska Institutet, Karolinska University Hospital, Solna, Stockholm, Sweden; 3 Department of Molecular Medicine and Surgery, Karolinska Institutet, Stockholm, Sweden; 4 Division of Obstetrics and Gynecology, Karolinska University Hospital Huddinge, Stockholm S-14186, Sweden

**Keywords:** Familial cancer, Uterine cancer, Lynch Syndrome, Multiple tumors

## Abstract

**Background:**

Uterine cancer (UC) represents 5.1% of all female malignancies in Sweden. Accumulation of UC in families occurs in around 5% of cases. We wanted to identify any familial association between UC and other selected cancers and to study the frequency of Lynch,Cowden and cancer syndromes among consecutive UC patients in Sweden.

**Methods:**

481 UC patients were included. Information on the cancer diagnoses of their relatives (first- (FDRs) and second-degree (SDRs) relatives and first cousins) was obtained. The relative frequencies of different cancers among relatives were compared to those in the Swedish general cancer population in 1970 and 2010. Families that fulfilled the criteria for hereditary cancer syndromes were tested for mutations in the causative genes. Families with at least one case of UC in addition to the index patient were compared to families with no additional cases to investigate possible characteristics of putative hereditary cancer syndromes.

**Results:**

There was an increased prevalence of UC in our study population compared to the Swedish general cancer population in 1970 and 2010 (6% vs. 4% and 3%, respectively). Seven families had Lynch Syndrome according to the Amsterdam II criteria. No families fulfilled the criteria for Cowden syndrome. In total 13% of index patients had at least one relative with UC and these families tended to have more cases of early onset cancer among family members. In addition, 16% of index patients were diagnosed with at least one other cancer. No families fulfilled the criteria for Cowden syndrome.

**Conclusion:**

We showed a familial clustering of UC among relatives of our index patients. Of the seven families with mutation-verified Lynch Syndrome, only one had been previously diagnosed, highlighting the need to increase gynecologists’ awareness of the importance of taking family history. Our data on multiple cancers and young age of onset in families with uterine cancer is compatible with the existence of additional hereditary uterine cancer syndromes.

## Background

Uterine cancer is the most common gynecological malignancy in Sweden. In 2010 it accounted for 5.1% of all female malignancies, with 1351 new cases [1]. Endometrial carcinoma accounts for the majority of uterine cancer, whereas uterine sarcomas are less common, accounting for less than 5% of all uterine cancer.

The risk of uterine cancer increases with age and the majority of cases are diagnosed in women aged 50–60 years [2,3]. The major risk factors for uterine cancer are diabetes mellitus, obesity, hypertension, polycystic ovary syndrome-anovulation, nulliparity and exposure to exogenous estrogens [2-4]. Most cases of uterine cancer are sporadic, however accumulation of endometrial cancer in families occurs in around 5% of cases [5].

Lynch syndrome (LS) is also known as hereditary non-polyposis colorectal cancer, where the female patient is also at high risk of developing endometrial cancer. LS has an incidence of 1:2000 – 1:660 [6]. LS is an autosomal dominant disorder caused by defective DNA mismatch repair genes (*MLH1, MSH2, MSH6* or *PMS2*) [7,8]. About 2% of uterine cancer is attributable to LS and among affected women aged younger than 50 years the prevalence of LS increases to 9% [9]. Women with LS have a 40-60% lifetime risk of developing endometrial cancer (equal to the risk for colorectal cancer) and about half of these women are diagnosed with endometrial cancer before colorectal cancer [10]. The median age of onset for endometrial cancer in women with LS is 46–62 years. These women are also at increased risk for gastric, ovarian, small intestine, urethral cancers, cancer of the hepatobiliary tract (part of liver and bile duct), skin (sebaceous gland tumours) and brain [3-11].

Cowden syndrome, an autosomal dominant disorder characterized by multiple hamartomas in the breast, thyroid and endometrium has a worldwide incidence of 1:250,000. Cowden syndrome is caused by a germline mutation in the *PTEN* gene and women with this mutation have a 5-10% lifetime risk of developing endometrial carcinoma [12]. A recent study concluded that 5% of serous uterine cancer was caused by mutations in *BRCA1, TP53* or *CHEK2 *[13], suggesting that some rare types of uterine cancer may have a family history consistent with hereditary breast and ovarian cancer syndrome (HBOC).

Several studies have investigated familial association in endometrial cancer. Women with a family history of endometrial cancer in their first-degree relatives have an increased risk of developing the disease, especially at a younger age (<55 years) [14,15]. A history of colorectal or ovarian cancer in FDRs has also been associated with an increased risk of endometrial cancer [15,16]. Uterine cancer may also be associated with a personal, or family history of breast cancer [17]. Despite these studies, genetic counseling is currently not offered to uterine cancer patients in Sweden, and only women with diagnosed LS or Cowden syndrome are referred for gynecological follow-up.

The aim of the present study was to investigate the prevalence of familial uterine cancer in Stockholm County, Sweden. We aimed to examine the existence of hereditary uterine cancer both related to LS or Cowden syndrome, and independent of these syndromes. Moreover, we investigated the possible associations between uterine cancer and family history of other cancers.

## Material and methods

All women from Stockholm County, Sweden with newly diagnosed uterine cancer are referred to the Department of Obstetrics and Gynecology, Karolinska University Hospital, Stockholm, Sweden. Uterine cancer patients who underwent surgery between January 2008 and March 2012 were invited to participate in the present study. Those who accepted (index patients) completed two questionnaires: one regarding diagnoses and age of onset of colorectal, breast, ovarian and other cancers in their family (i.e., the index patient, her first- and, second-degree relatives and first cousins), and a second questionnaire on their own risk factors (parity, history of diabetes mellitus, use of hormone replacement therapy, lipid lowering drugs, weight, height and former cancer diagnoses). Information from the second questionnaire was supplemented with information from the index patient’s records when necessary. At the end of the study period in 2012, the status of index patients was verified through the Swedish Cancer Registry, and updated when appropriate.

At enrollment all index patients provided a blood sample and DNA was extracted according to standard procedures for biobanking in the Registry of Endometrial Cancer in Stockholm, Sweden. Histopathological reports were obtained for all patients. Telephone interviews were conducted to get information for first- and, second-degree relatives and first cousins of the index patients. For all relatives with cancer, current age, or age at death, type of cancer and age at cancer diagnosis were recorded. Histological verification of cancer diagnoses in relatives was obtained from the Swedish Cancer Registry, medical records and/or death certificates.

Pedigrees were constructed for each index patient based on the information provided in the questionnaires and the telephone interview. All pedigrees were evaluated for the possible presence of hereditary uterine cancer syndrome according to the Amsterdam II criteria for LS [18] and the National Comprehensive Cancer Network guidelines for Cowden syndrome [12]. Families that fulfilled the criteria for HBOC were also identified [17]*.* Pedigrees were evaluated with a special focus on putative hereditary endometrial cancer, but colorectal, breast and ovarian cancer were also included in the evaluation. Screening for mutations in *MLH1, MSH2*, and *MSH6* for LS and *BRCA1* and *BRCA2* for HBOC was performed according to standard procedures. For the family analysis (Table 1), each index patient could only belong to one family, and only the side of the family with the most cancers was included in the analysis. If a relative had multiple primary cancers, they were each counted individually.

**Table 1:** Family history of cancer among the 481 index cases

**Heredity**	**N**	**(%)**
**At least 3 first-degree-relatives with any cancer**	38	(8)
**At least 2 first-degree-relatives with any cancer**	113	(24)
**At least 1 first-degree relative with any cancer**	284	(59)
At least 1 first-degree-relative with breast cancer OR at least 2 relatives* with breast cancer	82	(17)
At least 1 relative* with endometrial cancer	64	(13)
**At least 1 first-degree-relative with endometrial cancer**	33	(7)
At least 1 first-degree-relatives with colorectal cancer OR at least 2 relatives* with colorectal cancer	57	(12)
At least 1 relative* with ovarian cancer	29	(6)
At least 1 relative* with any cancer diagnosed at <50 years old	137	(29)

*Relatives comprise first- and second-degree relatives and first cousins. Only the side of the family with the most cancers was included in the analyses.

### Ethics statement

The study was approved by the Ethics Committee of Karolinska Institutet/ Karolinska University Hospital (DNR 2010/1536-31/2). Written informed consent was given by all participating women. Histological verification of cancer diagnoses of the relatives to the index patients was obtained with written consent of the relative, or if deceased, of their closest living relative.

### Statistics

The population-based Swedish Cancer Registry, which was started in 1958, was used as a reference population (general cancer population) to compare with the relative proportion of cancer diagnoses in relatives to our index patients. All physicians and pathologists are required to report all new cancers to this registry. The International Classification of Diseases Revision 7 (ICD-7) was used to classify all cancers apart from neoplasms of the lymphatic and hematopoietic tissue, where the ICD-8 classification system was used. The relative proportions of the different types of cancer in our cohort were compared to the relative proportions in the reference population from two different time points: 1970 and 2010. In 1970, Sweden had 8.08 million inhabitants, 28,594 of whom were reported to the cancer registry. In 2010, the corresponding figures were 54,342 and 9.4 million. In 1970 there were 2.621.732 inhabitants of an age >50 years, while in 2010 the corresponding number was 3.492.146. This comparison was done in order to compensate for differences in cancer incidence rates over time. The population data was weighted by age and gender of the cases. When age or gender was missing for relatives, the distribution of these variables was assumed to be the same as that among relatives with known age and gender. The number of observed cancers in relatives was assumed to be binomially distributed for each cancer site, with the total number of cancers defined as “number of trials”. Using binomial distribution, 95% confidence intervals (CIs) were calculated separately for each cancer site, without correcting for multiple testing. Site-specific CIs were transformed from numbers to proportions by dividing the number of cancers in relatives at a specific site by the total number of cancers in relatives. The CIs for the proportions were then compared with the proportions in the general cancer population in 1970 and 2010. Cancers with a significant difference in proportion compared with the general cancer population were considered over- or underrepresented.

Chi-square tests were used to test for heterogeneity in tables with categorical data. Missing/unknown values were excluded in chi-square tests unless otherwise indicated. P-values were calculated using Monte Carlo simulation in the chi-square tests. The Wilcoxon rank sum test detects shifts in distribution between groups, and was used for ordered outcomes. All calculations were performed in R (R Core Team, 2012).

## Results

Eight hundred ninety uterine cancer patients were invited, and 481 (54%) consented to participate in the study. Histopathological data was obtained from the Swedish Cancer Registry or medical records for all index patients and was available for the majority of cases in the relatives.

The median follow-up period for index patients was 24 months. The median age at diagnosis of index patients was 67 years (range 34–95 years). Histological examination revealed endometrioid carcinoma in 82% of the index patients, and most tumors (86%) were confined to the uterus (International Federation of Gynecology and Obstetrics-FIGO stage 1) (Table 2). During follow-up, 17 index patients (3.5%) had recurrent disease (median age 70.5 years); 12% were originally diagnosed with sarcomas, 6% had clear cell carcinomas, and 7% had endometrioid carcinoma. Of the index patients with recurrent disease, 12% were originally diagnosed as having stage 3 or 4 cancer (compared to 7% of the entire cohort) and 47% had a low-grade differentiation (compared to 22% in the entire cohort) (data not shown).

**Table 2:** Characteristics of the 481 index patients

**Characteristics**	**Number/total**	(%)*	**Median**	**Range [Min, Max]**
**Age at diagnosis, years**			67	[34, 95]
**Body mass index at diagnosis**			26.3	[17.6, 55.1]
**Hormone replacement therapy**	239/452	(52.9)		
**Parity**			2	[0, 8]
**Diabetes mellitus**	51/462	(11)		
**Lipid lowering drugs**	102/455	(22.4)		
**Histology**				
Endometrioid	394/481	(81.9)		
Serous or mixed	56/481	(11.6)		
Clear cell	9/481	(1.9)		
Sarcoma	20/481	(4.2)		
Hyperplasia with atypia	2/481	(0.4)		
**FIGO stage**				
1A	316/480	(65.8)		
1B	95/480	(19.8)		
2	34/480	(7.1)		
3A	16/480	(3.3)		
3B	7/480	(1.5)		
3C	2/480	(0.4)		
4	3/480	(0.6)		
4B	7/480	(1.5)		
**Grade**				
1	193/480	(40.2)		
2	181/480	(37.7)		
3	106/480	(22.1)		
**Depth of myometrial invasion**				
None	64/481	(13.3)		
<50%	282/481	(58.6)		
≥50%	128/481	(26.6)		
Through the serosa	7/481	(1.5)		
**Relapse**	17/481	(3.5)		

### Proportion of different cancer types in relatives

The relative proportions of different cancer types in the relatives of index patients were studied and compared to the general cancer population in 1970 and 2010. In total we found 1316 cancers in the relatives of index patients, 73 (6%) of which were uterine cancers. This was higher than the proportion of uterine cancer in the general cancer population in 1970 and 2010 (4% and 3% respectively) (Table 3). A similar overrepresentation of uterine cancer was identified among first-degree relatives alone, and among first- and second-degree relatives combined (data not shown).

**Table 3:** Proportion of different cancer types in relatives

	**Cancer site**	**Observed number**	**Proportion [%]**	**LL 95%**	**UL 95%**	**Proportion [%] in Sweden 1970**	**Proportion [%] in Sweden 2010**	**Reference outside CI**
1	Breast	207	15.73	13.75	17.71	14.57	19.84	No
2	Unspecified	140	10.64	8.97	12.31	3.29	2.26	CI above reference
3	Stomach/unspecified abdomen	127	9.65	8.05	11.25	7.04	1.38	CI above reference
4	Prostate	115	8.74	7.22	10.26	7.89	13.21	No
5	Colon	109	8.28	6.84	9.8	7.75	6.81	No
6	Lung and airways	81	6.16	4.86	7.45	5.89	6.26	No
7	Uterus	73	5.55	4.33	6.84	3.73	3.05	CI above reference
8	Biliary passages and liver	49	3.72	2.74	4.79	3.27	1.54	No
9	Cervix	45	3.42	2.51	4.41	3.73	1.26	No
10	Brain and nervous system	45	3.42	2.51	4.41	3.47	3.01	No
11	Rectum	34	2.58	1.75	3.5	3.96	3.64	CI below reference
12	Pancreas	32	2.43	1.67	3.27	3.14	1.66	No
13	Ovary/fallopian tube	30	2.28	1.52	3.12	4.11	1.79	No
14	Kidney	28	2.13	1.37	2.96	3.71	1.86	No
15	Malignant melanoma	28	2.13	1.37	2.96	2.18	5.83	No
16	Other skin cancer	24	1.82	1.14	2.58	2.32	7.45	No
17	Leukemia	23	1.75	1.06	2.51	2.56	2.31	No
18	Urinary tract	20	1.52	0.91	2.2	3.64	3.77	CI below reference
19	Larynx	15	1.14	0.61	1.75	0.51	0.29	CI above reference
20	Hodgkin’s disease	12	0.91	0.46	1.44	0.94	0.41	No
21	Multiple myeloma	12	0.91	0.46	1.44	1.25	1.09	No
22	Bone	11	0.84	0.38	1.37	0.25	0.14	CI above reference
23	Non-Hodgkin lymphoma	11	0.84	0.38	1.37	2.27	2.99	CI below reference
24	Thyroid	10	0.76	0.3	1.29	1.02	1.11	No
25	Esophagus	9	0.68	0.3	1.14	0.79	0.6	No
26	Connective tissue	9	0.68	0.3	1.14	0.7	0.53	No
27	Lip/tongue/mouth	6	0.46	0.15	0.84	1.18	0.98	CI below reference
28	Vulva/vagina	3	0.23	0	0.53	0.56	0.43	No
29	Testis	3	0.23	0	0.53	0.4	0.75	No
30	Endocrine glands (excl thyroid)	2	0.15	0	0.38	1.45	1.32	CI below reference
31	Polycytemia vera	2	0.15	0	0.38	0.39	0.24	No
32	Penis/scrotum	1	0.08	0	0.23	0.18	0.11	No
33	Salivary gland	0	0	0	0	0.28	0.21	CI below reference
34	Pharynx	0	0	0	0	0.44	0.6	CI below reference
35	Small intestine, including duodenum	0	0	0	0	0.42	0.48	CI below reference
36	Peritoneum	0	0	0	0	0.02	0.14	CI below reference
37	Nose, nasal cavities, middle ear and accessory sinuses	0	0	0	0	0.28	0.12	CI below reference
38	Mediastinum	0	0	0	0	0.02	0.01	CI below reference
39	Eye	0	0	0	0	0.32	0.25	CI below reference
40	Myelofibrosis	0	0	0	0	0.03	0.27	CI below reference

The proportion of observed cancer cases among first degree relatives, second degree relatives and first cousins to the index cases. The proportion is compared to the expected distribution of cases in Sweden according to statistics from the National Board of Health and Welfare. The expected distribution is adjusted for the age and sex in observed cases. CI: Confidence Interval.

Moreover, cancers of the stomach/unspecified abdomen, larynx and bone were also overrepresented in our relatives compared to the general cancer population in 1970 and 2010. However, there was no overrepresentation of the number of breast (n = 207, 16%), colon (n = 109, 8%), rectal (n = 34, 3%) or ovarian (n = 30, 2%) cancers in relatives. Cancers of the rectum, pancreas, urinary tract, non-Hodgkin lymphoma, lip/tongue/mouth, endocrine glands (excluding thyroid), pharynx, small intestine, peritoneum, nose, mediastinum, eye and myelofibrosis were present in a smaller proportion of relatives than in the general population (Table 3).

### Presence of hereditary cancer syndromes

**Figure 1:**
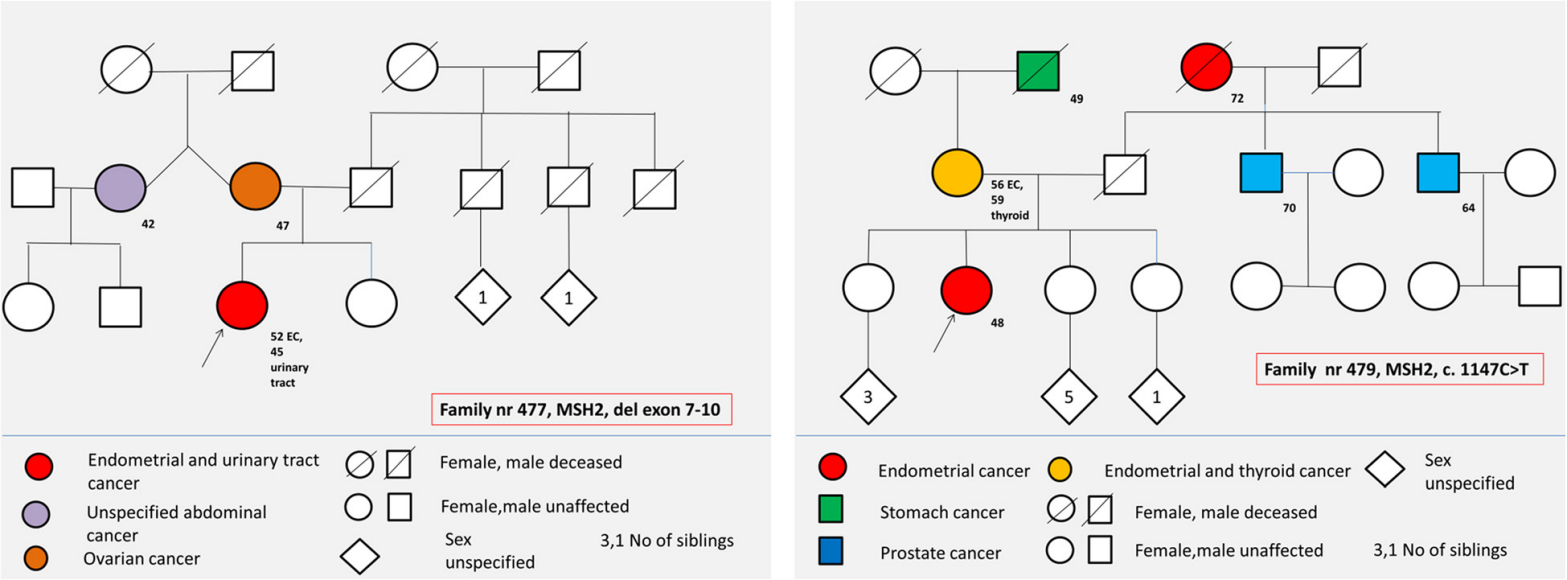
**Pedigrees of two families that were diagnosed with Lynch syndrome. ***MSH 2*, del exon 7–10 and *MSH2*, c1147C > T mutations were diagnosed as part of the study. Note that there was no known colorectal cancer in any of the families.

Pedigrees were evaluated for possible hereditary cancer syndromes, in order to perform genetic testing and to refer the patient for genetic counseling. Nine index patients (9/481, 2%) fulfilled the Amsterdam II criteria for LS. All nine had endometrioid carcinoma diagnosed at a median age of 58 years (range: 39–80). Seven of the nine had deleterious mutations in mismatch repair genes: three in *MLH1* (c.546-2A > G; c.790 + 1G > C and deletion of exon 1–3) and four in *MSH2* (c.1147C > T; c.1786_1788del; deletion of exon 7–10 and deletion from exon 3 of the *EPCAM* gene to exon 6 of *MSH2*). One kindred was a known LS family, the other six were diagnosed as part of this study. Two of the index patients with LS had no known family history of colorectal cancer (Figure 1). No index patients fulfilled the National Comprehensive Cancer Network guidelines for Cowden syndrome and none were screened for mutations in the *PTEN* gene.

Nine pedigrees fulfilled the testing criteria for HBOC. Only one of the nine index patients had serous carcinoma, two had uterine sarcoma and six had endometrioid carcinoma. Six families were screened for mutations in *BRCA1* and *BRCA2*, but no mutations were found. In the three remaining families, the index patient did not have breast or ovarian cancer and declined genetic counseling for further genetic investigation of the family.

### Family history of cancer

Eight percent of all index patients had at least three first-degree relatives with a cancer diagnosis, 24% had at least two, and 59% had at least one (Table 1). As uterine cancer may be associated with colorectal, ovarian and breast cancer, families where the index patient had at least one first-degree relative or at least two relatives with these diagnoses were also assessed. According to these criteria, 17% of our index patients had relatives with breast cancer, and 12% had relatives with colorectal cancer. As ovarian cancer is rarer, all families where the index cases had at least one relative with ovarian cancer were counted (n = 29; 6%) (Table 1).

In total, 64 index patients had at least one relative with uterine cancer (13%). These families were compared to the 417 families of index patients who had no relatives with uterine cancer (Table 4). The two groups of index patients had very similar distributions of histology and other characteristics, such as age at diagnosis, stage, relapse, ploidy and presence of multiple cancers. 47% (30/64) had relatives diagnosed with cancer before the age of 50 years, compared to 26% (107/417) in the group of index patients with no relatives with uterine cancer (p < 0.001) (Table 4). Four of the thirty families with relatives with uterine cancer had LS. In twelve of the 64 families, at least one relative or index case was diagnosed with uterine cancer before 50 years of age. Two of these families had LS.

**Table 4:** Comparison of the characteristics and variables in families with and without additional cases of uterine cancer

		**Uterine cancer in family n = 64**		**No Uterine cancer in family n = 417**		
		**Median**	**Range**	**Median**	**Range**	**P-value**
**Age (years)**		65	(36,9)	67	(35,0)	0.082 (Wilcoxon rank sum test)
**Body mass index**		26.6	(17.6,43.4)	26.2	(17.7,55.1)	0.466 (Wilcoxon rank sum test)
		**number**	**row percent**	**number**	**row percent**	
**Histology**	**Endometrioid**	52	(81%)	342	(82%)	0.102 (chi.sq test)
	**Serous or mixed**	4	(6%)	52	(12%)	
	**Clear cell**	3	(5%)	6	(1%)	
	**Sarcoma**	5	(8%)	15	(4%)	
	**Hyperplasia**	0	(0%)	2	(0%)	
**FIGO stage**	**1A**	45	(70%)	271	(65%)	0.367 (chi.sq test)
	**1B**	11	(17%)	84	(20%)	
	**2**	2	(3%)	32	(8%)	
	**3A**	4	(6%)	12	(3%)	
	**3B**	0	(0%)	7	(2%)	
	**3C**	0	(0%)	2	(0%)	
	**4**	0	(0%)	3	(1%)	
	**4B**	2	(3%)	5	(1%)	
**Relapse**	**No**	61	(95%)	403	(97%)	0.726 (chi.sq test)
	**Yes**	3	(5%)	14	(3%)	
**Ploidy**	**Aneuploid**	13	(22%)	108	(28%)	0.338 (chi.sq test)
	**Diploid**	47	(78%)	274	(72%)	
**Multiple cancers**	**No**	52	(81%)	354	(85%)	0.478 (chi.sq test)
	**Yes**	12	(19%)	63	(15%)	
**Uterine cancer <50 years of age**	**No**	57	(89%)	389	(93%)	0.300 (chi.sq test)
	**Yes**	7	(11%)	28	(7%)	
**Relative with cancer <50 years of age**	**No**	34	(53%)	310	(74%)	0.001 (chi.sq test)
	**Yes**	30	(47%)	107	(26%)	

Six families had at least two cases of uterine cancer with no other concurrent cancers and may possibly represent site-specific uterine cancer families. In three of these families, the index patient was diagnosed before the age of 50 years.

### Presence of multiple cancers in index patients

Seventy-five index patients (16%) had at least one cancer in addition to their uterine cancer. Thirty-four of them had breast cancer (one woman had bilateral disease), of whom 31 were diagnosed before their uterine cancer, two were diagnosed at the same time, and two had uterine cancer prior to their breast cancer diagnosis. The median time difference between breast and uterine cancer diagnosis was 7 years. Twelve index patients with breast cancer had previously used selective estrogen receptor modulators. Histological examination of the uterince cancers showed endometrioid carcinoma in 80%, 6% each of serous carcinoma, clear cell carcinoma or sarcoma, and mixed type in 3%.

Fourteen index patients had colorectal cancer in addition to uterine cancer. Twelve had a diagnosis of colorectal cancer prior to their diagnosis of uterine cancer, one at the same time, and one after diagnosis of uterine cancer. The median time between previous colorectal cancer and uterine cancer was 6 years. Of the uterine cancers in this group, 86% were endometrioid carcinoma, 7% were serous carcinoma and 7% were clear cell carcinoma.

Ovarian cancer was diagnosed synchronously with uterine cancer in four index patients. Three of these women had endometrioid carcinoma and one had serous carcinoma. Moreover, nine index patients had more than two cancers: seven index patients with breast and uterine cancer had additional cancers, including colorectal cancer, myeloma, skin cancer, salivary gland cancer and malignant melanoma. In addition, two index patients with uterine and colorectal cancer also had urinary tract or skin cancer. In total, four index cases with multiple cancers had LS: one had colorectal cancer, one had colorectal cancer and urinary tract cancer, one had skin cancer, and one had urinary tract cancer in addition to uterine cancer (Table 5).

**Table 5:** Multiple tumors in 75 of the 481 index cases

**Additional tumors**	**Of patients with multiple tumors**	**Of total patient population**	**Nr in group with other tumors**	**Other tumors$**
Breast cancer (BR)	34/75 (45%)§	34/481 (7%)	7/34	CRC + myeloma, CRC + skin, CRC (2 patients), salivary gland, malignant melanoma, skin
Colorectal cancer (CRC)	14/75 (19%)	14/481 (3%)	6/14	*BR + myeloma*, *BR + skin*, *BR (2 patients)*, urinary tract, skin
Ovarian cancer	4/75 (5%)	4/481 (0.4%)	0/4	
Other cancer*	34/75 (45%)	34/481 (7%)	7/34	*Skin (3 patients)*, *myeloma*, *salivary gland*, *malignant melanoma*, *urinary tract.*

Note that some patients had more than one additional tumor.

§12/34 (35%) had been treated with selective estrogen receptor modulator therapy.

*11 with skin cancer; 5 malignant melanoma, 4 cervical cancer, 4 lymphoma, 2 myeloma, 2 urinary tract tumors (UT), 1 each of: kidney, central nervous system, lung, extra-adrenal paraganglioma, salivary gland and vocal cord cancer.

$Diagnoses in italics have already been mentioned in the columns above.

## Discussion

This study investigated family history of cancer in an unselected group of uterine cancer patients in Sweden. Our study population included 481 consecutive patients with uterine cancer and their close relatives. The main finding of the present study is that uterine cancer is associated with a family history of uterine cancer: 13% of our index patients had at least one relative with the same disease, and 7% of these had at least one FDR with uterine cancer. We also found an increased relative proportion of uterine cancer in families from our cohort compared with the observed proportions in the general cancer population for the years 1970 and 2010.

Cancer diagnosis before the age of 50 years and multiple cancers may also be indicative of a hereditary cancer syndrome. Almost half of the families with at least two cases of uterine cancer included relatives diagnosed with cancer before the age of 50 years (n = 30) and LS was the cause in only four (13%). Also 17% of our index patients had multiple cancers. The present results agree with and extend previous findings on familial uterine cancer, which showed that first-degree relatives of patients with endometrial cancer have an increased risk of developing the same cancer (with odds ratios between 1.5 and 2.8). The higher odds ratios were found in relatives of uterine cancer patients diagnosed before the age of 55 years [14-21].

Seger et al. [22] reported that environmental factors interact with genetic susceptibility. In that study the risk of endometrial cancer increased, not only with the degree of the familial relationship, but also with the participant’s body mass index (BMI). Thus first-degree relatives of obese endometrial cancer patients had a relative risk (RR) of 3 of developing endometrial cancer compared to first-degree relatives of patients with normal/low BMI, who had a RR of 1.12. However, in the present study, there was no difference in BMI (median 26.6) between families with or without additional uterine cancer cases.

In the present study, there was an increased relative proportion of laryngeal, stomach/abdominal and skeletal cancer. One explanation for this excess could be misclassification of metastasis (for skeletal cancer), possible differential recall/information/classification of cases (especially stomach/abdominal cancer). Currently there is no known common genetic and/or environmental factor that can explain a possible association between uterine cancer and laryngeal cancer.

Surprisingly, we found no overrepresentation of breast cancer in our cohort compared to the general cancer population. In a similar analysis, endometrial cancer was overrepresented in 803 non-*BRCA1/2* breast cancer families compared to the general cancer population [17]. The authors suggested that endometrial cancer and breast cancer could constitute a new breast cancer syndrome. In our study, 45% of our index patients with multiple primary cancers had uterine cancer and breast cancer. This is higher than the 31% found by Delin et al. [23] or the 10% found by Uccella et al. [24], and suggests that there might be an association between these two tumors. Other studies have also found that a personal history of breast cancer increases the risk of endometrial cancer regardless of family history [25]*.* Breast cancer was especially associated with a risk of developing subsequent serous carcinoma in younger women [26]. Pennington et al. [13] recently reported on a study population in which seven (5%) of women with serous carcinoma had mutations in breast cancer genes, but only two of the seven had breast cancer in their family history. Only 6% of our index patients had serous carcinoma and breast cancer, and no *BRCA1/2* mutations were found in families that fulfilled the testing criteria for HBOC.

Tamoxifen treatment is a known risk factor for endometrial cancer with an RR of 2.2-4, especially in postmenopausal women [27,28]*.* The cumulative incidence of endometrial cancer five years after tamoxifen treatment was 13/1000 compared to 5.4/1000 in women who did not use tamoxifen [27]. Thus, development of uterine cancer after breast cancer cannot be solely attributed to tamoxifen therapy in the 12 index patients in our study who used it.

Ovarian cancer was not overrepresented in our study; only 5% of our index patients with multiple primary cancers had ovarian cancer, which is similar to the 4% reported by Uccella et al. [24], but lower than the 29% reported by Delin et al. [23]. Studies on familial coupling of ovarian and uterine cancer have shown contradictory results [14-16]. However, Hemminki et al. [29] demonstrated a high risk of synchronous or metachronous ovarian and endometrial cancer, especially endometrioid carcinoma. Our study population is too small to draw any conclusions on an association between ovarian and uterine cancer.

Our study showed no overrepresentation of colorectal cancer either, despite the well-known association between uterine cancer and colorectal cancer in LS. However, 17% of our index patients with multiple primary cancers had colorectal cancer. This is higher than the 3% reported by Uccella et al. [24], but similar to the 11% reported by Delin et al. [23], which was more than the percentage of expected colorectal cancers in the female population in their study among our 14 index patients with metachronous colorectal cancer, only two had LS.

In the present study, nine families (1.9%) fulfilled the Amsterdam II criteria and of these seven (1.5%) had mutation-verified LS: three with mutations in *MLH1* and four in *MSH2*. This correlates well with other studies on unselected uterine cancer cases, which have found LS in 1.8-4% [11,30-32], while LS was found in 9% of uterine cancer cases diagnosed before the age of 50 years [10]. In these five studies, a total of 44 LS families were identified. Thirty-six percent had mutations in *MSH6* and 39% in *MSH2. MSH6* mutation carriers are less likely to meet Amsterdam II criteria [33] and have a lower risk of colorectal cancer (10-22% cumulative risk by 70 years of age) and of other LS-related cancers [34,35]. As the Amsterdam II criteria have a reported sensitivity of 60-80% for colorectal cancer [36], but of only 20-30% in consecutive endometrial cancers [11,30], it is likely that our selection criteria have missed a number of LS families, especially those caused by *MSH6* mutations. Thus the 1.5% of LS cases in our study is likely to be an underestimate. A small number of families with two or more cases of uterine cancer may also represent site-specific uterine cancer distinct from LS.

## Conclusions

We found an overrepresentation of uterine cancer among first- and second- degree relatives and first cousins of uterine cancer patients. This phenomenon could be explained by a common genetic factor and/or common environmental, life-style factors and further studies are needed to confirm this. In addition, our data on multiple cancers and young age of onset in families with uterine cancer is compatible with the existence of additional hereditary uterine cancer syndromes. The prevalence of LS was about 2% in our consecutive population of uterine cancer patients. Only one of seven mutation-verified LS families had been previously diagnosed, although they demonstrate family history consistent with a high risk of LS. Alerting gynecologists of the increased risk of uterine cancer among close relatives and the prevalence of LS and other hereditary cancer syndromes is a feasible strategy to increase appropriate referral for further genetic counseling and investigation and better surveillance of individuals at high risk of uterine cancer.

## Competing interests

No part of these data have been sent or published elsewhere. None of the authors have any conflicts of interests.

## Authors’ contributions

GT conceived and designed the experiments analyzed and interpreted the data, drafted the manuscript. OW conceived and designed the experiments, analyzed and interpreted the data. ET conceived and designed the experiments, analyzed and interpreted the data, drafted the manuscript. KGD conceived and designed the experiments drafted the manuscript. AL conceived and designed the experiments drafted the manuscript. MM conceived and designed the experiments analyzed and interpreted the data. All authors read and approved the final manuscript.
